# Surgical technique: posterior cruciate ligament (PCL) reconstruction using double posteromedial arthroscopic portals

**DOI:** 10.1186/s40634-023-00698-6

**Published:** 2023-12-13

**Authors:** Gian Andrea Lucidi, Romain Seil, Piero Agostinone, Cécile Toanen, Alberto Grassi, Stefano Zaffagnini

**Affiliations:** 1 Clinica Ortopedica E Traumatologica II, IRCCS Istituto Ortopedico Rizzoli, Via Giulio Cesare Pupilli, 1, Bologna, Italy; 2 Department of Orthopaedic Surgery, Centre Hospitalier Luxembourg–Clinique d’Eich, 78 Rue d’Eich, Luxembourg, L‑1460 Luxembourg; 3 Sports Medicine and Science, Luxembourg Institute of Research in Orthopaedics, Luxembourg, Luxembourg; 4 Unit for Human Motion Orthopaedics, Sports Medicine and Digital Methods, Luxembourg Institute of Health, Luxembourg, Luxembourg; 5 Service de Chirurgie Orthopédique, Centre Hospitalier Départemental Vendée, La Roche-sur-Yon, France

## Abstract

The posterior cruciate ligament (PCL) reconstruction is a technically demanding surgical procedure that requires optimal identification of both the femoral and the tibial anatomical footprints. To aid the tibial tunnel placement and many authors recommend creating a posteromedial (PM) portal. The further addition of a second PM portal, which could be used as a “working portal”, may further allow a more straightforward reconstruction by improving the identification of the anatomical footprint, the clearing of the stump, and the graft passage.

## Bakground

Posterior Cruciate Ligament (PCL) tears are serious high-energy injuries with possible severe short and long-term consequences [1, 2]. An integrated diagnostic workup is mandatory to assess the injury’s severity and establish a correct treatment. The management of PCL injuries aims to restore native knee kinematics and function and avoid persistent posterior laxity and degenerative changes. While some PCL injuries could be managed nonoperatively, surgical reconstruction of the PCL may be indicated in symptomatic high-grade posterior laxity, multiligament injuries, or failed conservative management.

During PCL reconstruction, precise visualization and preparation of the PCL tibial footprint are essential to avoid tunnel malposition, difficulties in graft passage, and neurovascular damage.

For this purpose, some authors advocate using an additional 70° scope, while others prefer to perform a single posteromedial (PM) portal.

The decision to perform two posteromedial portals is related to several factors, including its relative safety. First of all, recent cadaveric studies have shown that the “safe” margin of the PM compartment is consistently wider than the posterolateral compartment and that the popliteal artery is located lateral to the posterior capsular septum [3, 4]. Moreover, other studies have shown that sex and age do not influence the width of the PM safe zone and that all the neurovascular structure are located at least 15 mm away, even when two PM portals are established [4, 5].

The present article aims to describe the technique and the possible advantages of PCL reconstruction performed using double PM portals [6].

## Surgical technique

The anesthetized patient is positioned with knee flexed at 90° to avoid vascular injury by distancing the popliteal artery from the femur and to allow correct positioning of the two PM portals by relaxing the posterior capsule and opening the PM compartment.

**Fig. 1:**
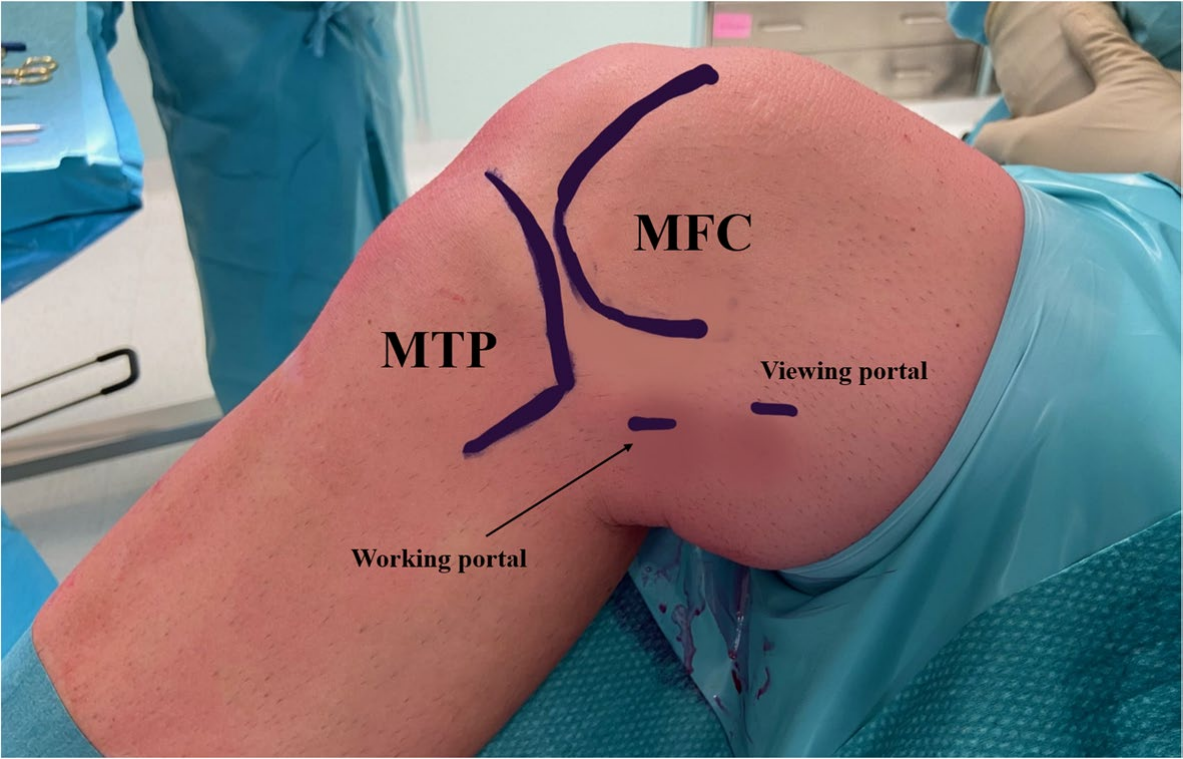
Anatomical landmarks for the identification of the two posteromedial portals position. MFC (medial femoral condyle); MTP (medial tibial plateau)

**Fig. 2:**
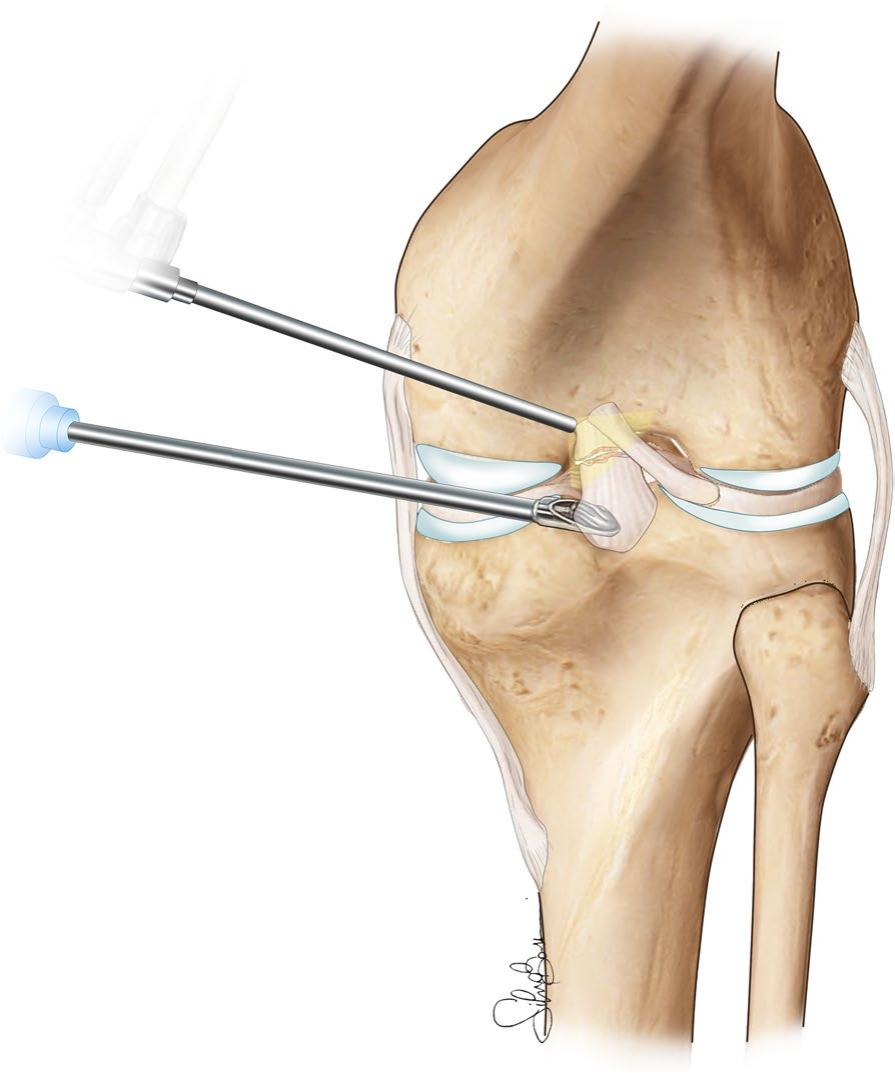
Graphical representation of the arthroscopic instrumentation inserted in the PM portals, note that the double access allows a better visualization and an easier approach to the PCL tibial footprint

A tourniquet is usually applied at the proximal thigh to improve visualization. The position of the two PM portals was identified thanks to three landmarks: the medial femoral condyle (MFC), the medial tibial plateau (MTP) and the joint line. The viewing portal is proximal to the medial femoral condyle and posterior to the posterior femoral cortex, and the working portal at the height of the joint line, 3–4 cm more distal (Figs. 1 and 2) [4].

Arthroscopic anterolateral (AL) and anteromedial (AM) portals are created close to the patellar tendon, as most surgical steps are performed within the intercondylar notch. If present, meniscal tears should be managed according to the patient’s and lesion’s characteristics.

At this point, the PCL should be carefully evaluated to confirm the indication for reconstruction. Indirect signs of PCL insufficiency include posterior tibial subluxation and anterior cruciate ligament (ACL) “pseudolaxity” [7]. Moreover, a probe could be used to palpate the PCL fibers to evaluate their tension while the assistant applies a posterior drawer.

Once the indication for PCL reconstruction has been confirmed, attention should be turned to the identification of the PCL tibial footprint for the creation of the tibial tunnel.

**Fig. 3:**
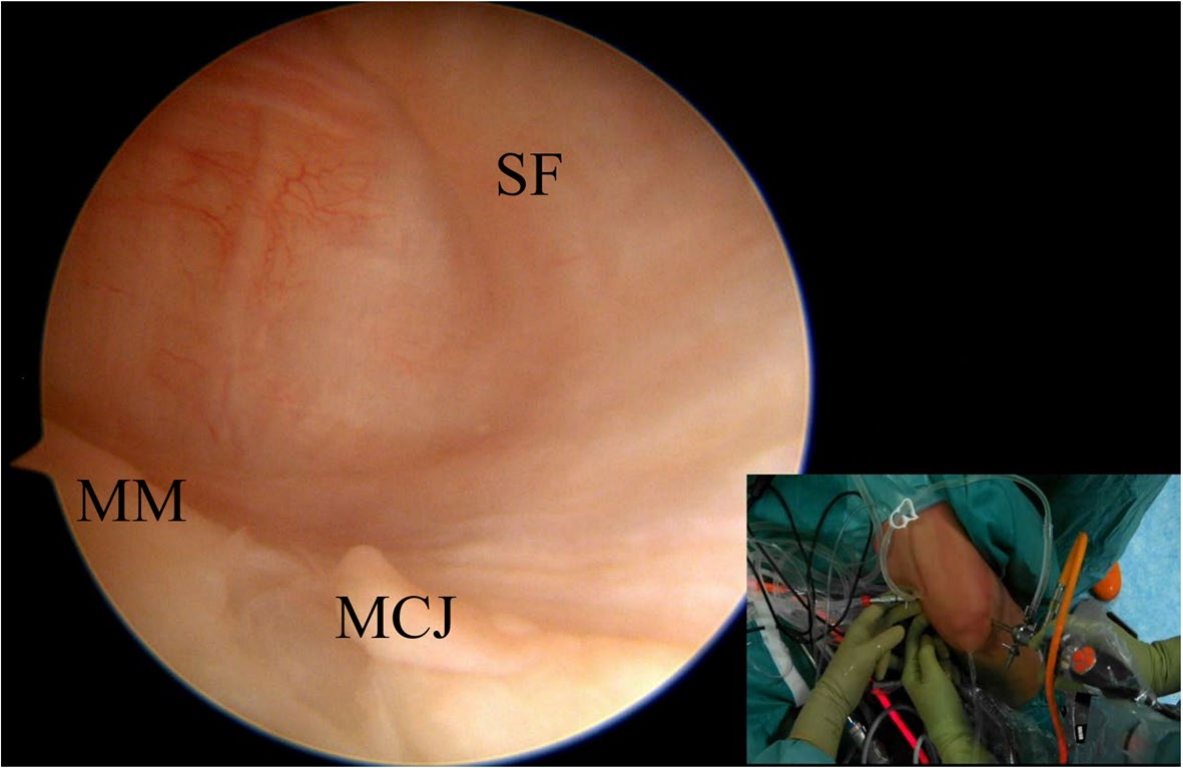
From the standard anterolateral portal, the arthroscope is advanced into the notch to evaluate the posterior posteromedial capsule. The knee flexed at 90° and the transillumination helps to prevent damage to the neurovascular structures. SF (synovial fold); MM (medial meniscus); MCJ (menisco capsular junction)

The 30° arthroscope introduced in the AL portal should be advanced through intercondylar notch and then in the posteromedial compartment to identify the “safe zone” where the two posteromedial portals could be created (Fig. 3) [3, 4]. Before the creation of the portals, it is essential to identify the space between the semimembranous and gastrocnemius fold and avoid being too inferior to protect the branches of the saphenous nerve [6].

### Creation of the first PM portal (viewing portal)

**Fig. 4:**
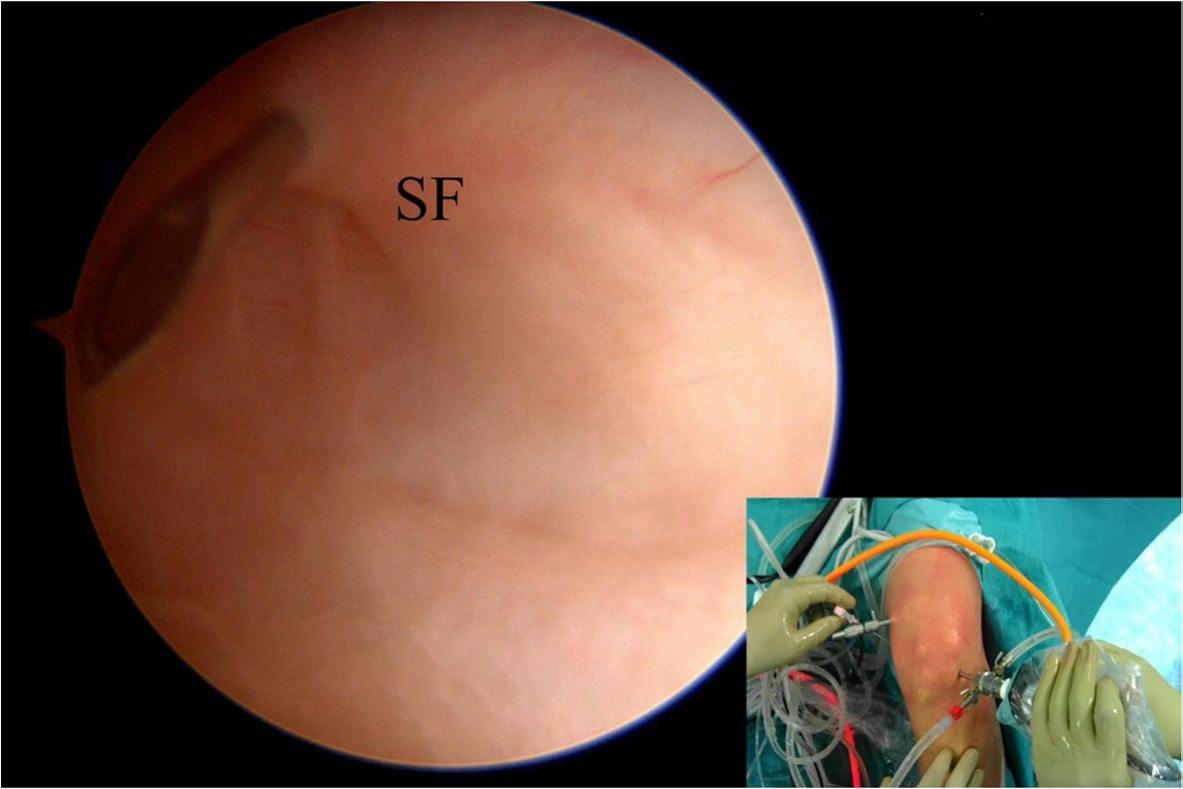
Under arthroscopic visualization via transnotch view, a needle is inserted to identify the position of the “first” posteromedial portal (viewing portal). The needle is inserted just proximal to the posteromedial capsular fold. SF (synovial fold)

A spinal needle (18 gauge) is inserted under arthroscopic visualization in the superior-posterior quadrant of the PM capsule (Fig. 4). The needle should be inserted slightly proximal or at the level of the synovial fold and directed downward.

Following the direction of the spinal needle and under arthroscopic control, an 11-blade scalpel is used to create the portal, which is then enlarged using a trocar placed on a switching stick. The camera could be then inserted in the first PM portal to identify the tibial PCL footprint, including the “shiny fibers” of the posterior horn of the medial meniscus that represent its anterior landmark [8].

### Creation of the second PM portal (working portal)

**Fig. 5:**
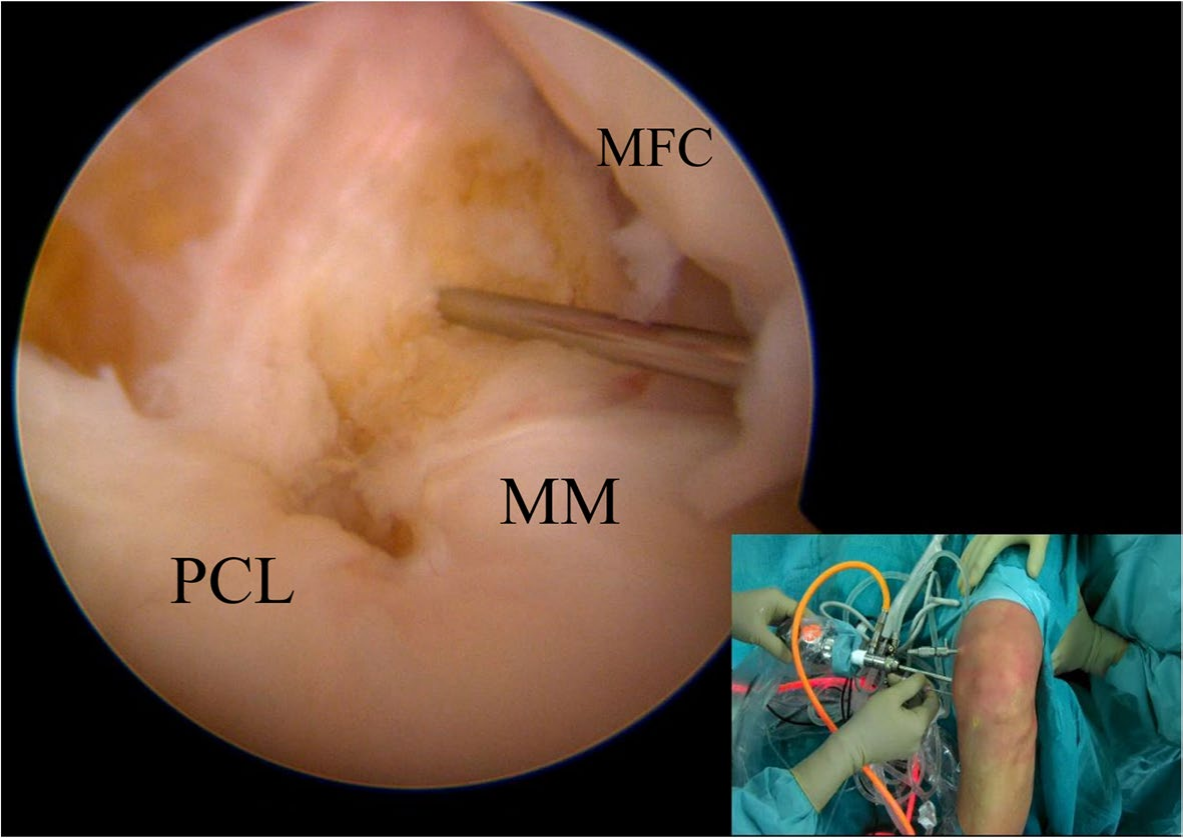
The arthroscope is inserted in the “first” posteromedial portal (viewing portal). Under direct visualization, a second needle is introduced just posterior and proximal to the edge of the medial tibial plateau to identify the correct position for the “second” posteromedial portal (working portal). MFC (medial femoral condyle); MM (medial meniscus); PCL (posterior cruciate ligament)

The second (working) PM portal could be created under arthroscopic control with the help of transillumination to reduce the risk of saphenous nerve and vein damage. This portal should be created about 3 cm distal and 2 cm anterior to the first PM portal, at the level of the posterior edge of the tibial plateau (Fig. 5) [4].

### Mistakes that may occur while placing the portals

- Excessively anterior position: the introduction of the instruments may be difficult because of the tibia. Moreover, finding the correct path for the instrument introduction may take time since the visualization could be impaired.
- Excessively low position: the vision could be challenging, and the instruments may be deflected against the posterior portion of the tibial plateau.
- Excessively high position: there will be an increased distance between the skin and the posterior capsule [9, 10].

### Tibial tunnel

**Fig. 6:**
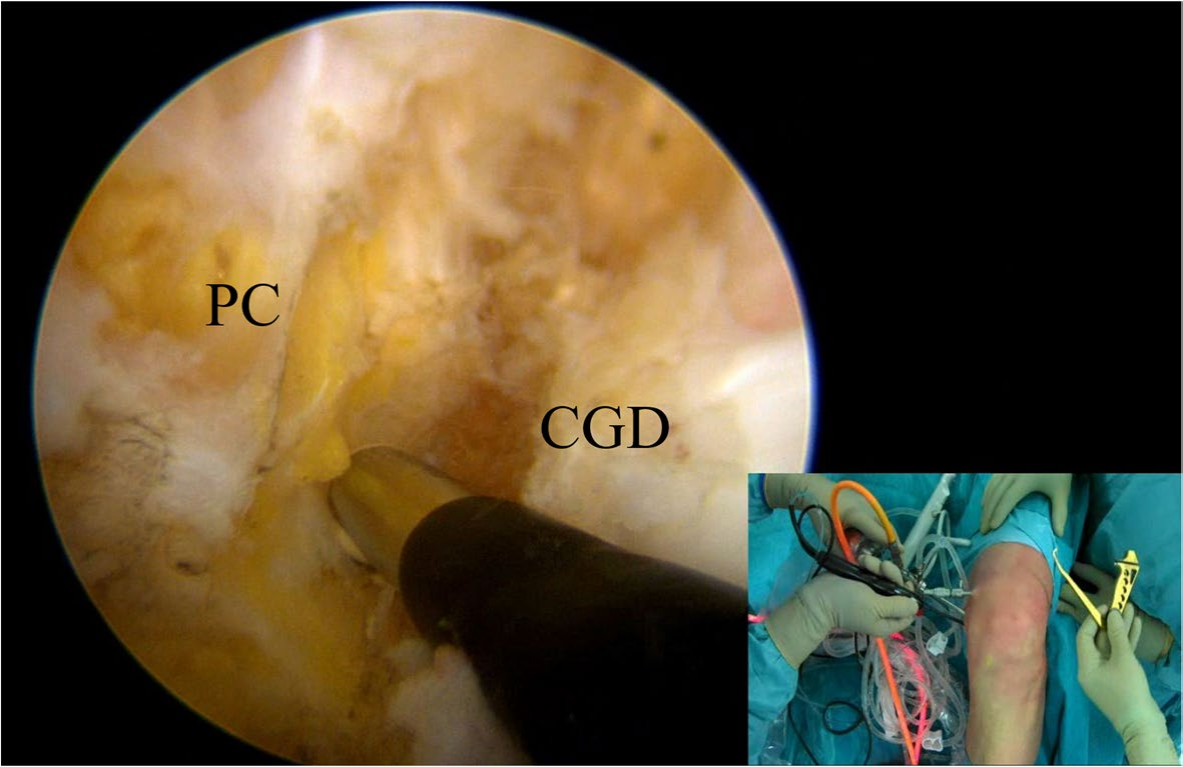
Under direct visualization from the “first” posteromedial portal, a radiofrequency ablation device is used to clean the tibial PCL footprint. Note that the ablator is inserted from the “second” posteromedial portal. PC (posterior capsule); CGD (Champagne-glass drop-off)

Under direct visualization through the “first” PM portal, a radiofrequency ablator is introduced into the PM compartment and it is used to identify and clean the tibial PCL footprint. At this point it is important to elevate the capsule and create a space of about 5–10 mm from the “champagne glass drop-off” of the tibia and the posterior capsule to visualize the exit point of the guide pin (Fig. 6).

With the knee flexed at 90°, a tibial PCL guide could be introduced into the AM portal and centered in the tibial PCL footprint. At the same time, the extra-articular point of the tibial PCL guide is located on the anteromedial tibia around 5–7 cm distal to the joint line and 2–3 cm medial to the tibial crest. Under arthroscopic control, the guide pin could be advanced into the bone, and once the correct position of the pin has been confirmed, it could be over-reamed with a 9-12 mm acorn reamer.

**Fig. 7:**
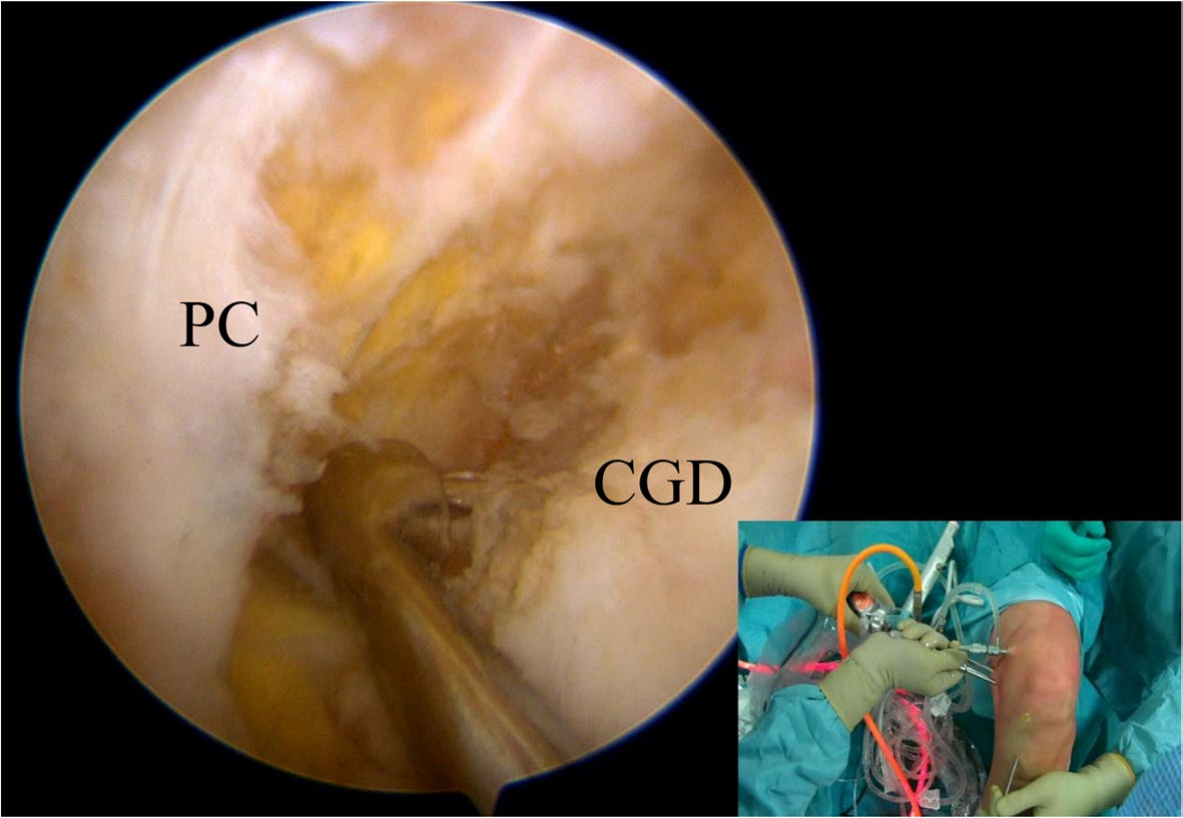
Under direct visualization from the “first” posteromedial portal, a curette introduced from the “second” posteromedial portal is used to protect the neurovascular structure during the tibial tunnel drilling. PC (posterior capsule); CGD (Champagne-glass drop-off)

At this point, a curette introduced from the “second” PM portal and placed at the tip of the guide pin could be used to avoid its posterior advancement and overpenetration (Fig. 7). Once the tibial tunnel has been correctly drilled, a shaver could be introduced into the tunnel and in the “working” PM portal to remove bony spicules and soft tissue that could impair the graft passage.

### Femoral tunnel

The femoral footprint of the PCL should be cleaned with an ablator to identify the anterolateral bundle attachment better. The trochlear and medial arch point of the medial femoral condyle defines the center of the AL bundle that should be marked with an ablator. Its position is located about 5 mm posterior to the chondral margin.

At this point, an additional “low” anterolateral instrument portal could be created to aid in the femoral tunnel creation. A needle could be used to define better the ideal position: a too-lateral portal may cause damage to the lateral femoral condyle cartilage, while a too-medial one can damage the infrapatellar fat pad and create a sharp angle with the femur [10, 11].

Once the correct position of the AL bundle has been marked, a guide pin could be drilled all the way through the medial condyle with the knee flexed at 90°.

At this point, the guide pin is completely over-reamed with a 4.5 mm drill first and then with a 9-11 mm reamer to create a half-tunnel on the medial femoral condyle [12]. Then, a shaver is used to remove soft tissue remnants and bone debris from the tunnel to facilitate graft passage and a passing suture.

### Graft passage and fixation

The tibial suture shuttle is passed first under arthroscopic control from the PM viewing portal. An awl with a suture shuttle is introduced through the tibial tunnel and is retrieved with a grasper from the high AM portal.

**Fig. 8:**
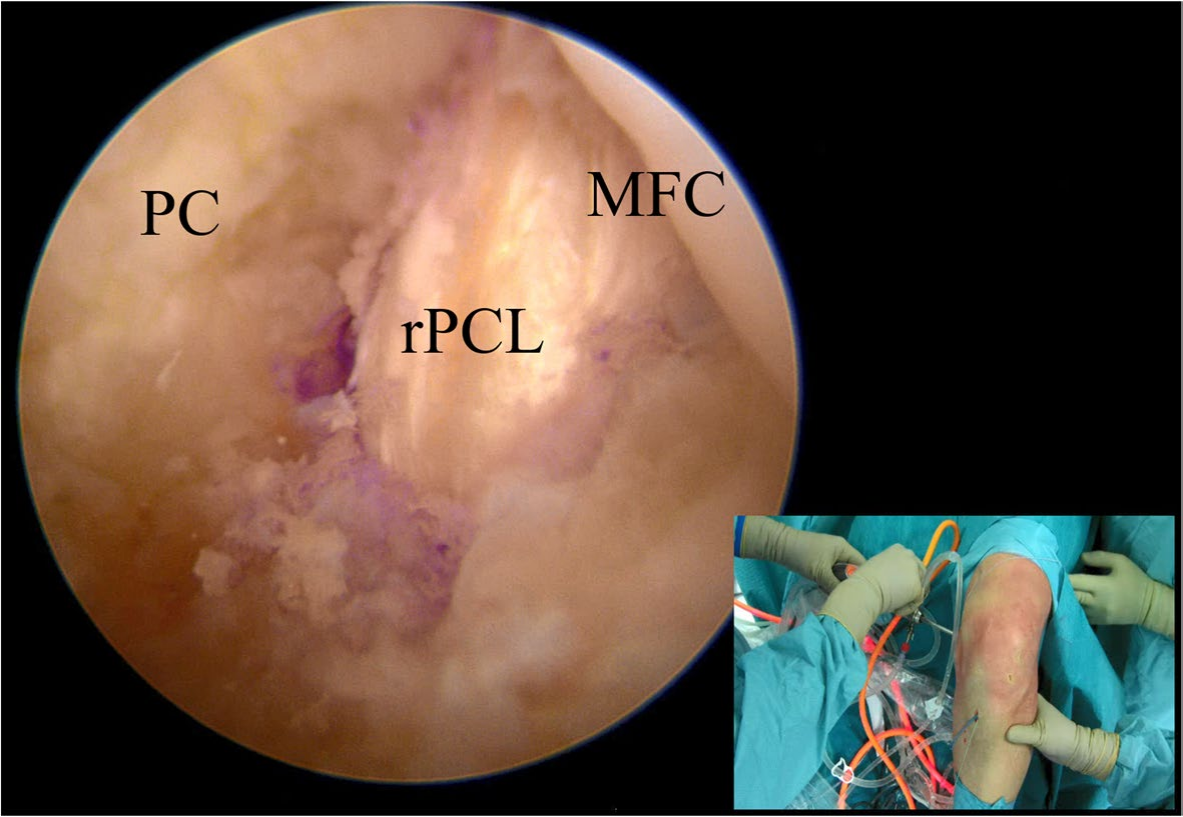
Visualization of the reconstructed PCL through the viewing portal. PC (posterior capsule); MFC (medial femoral condyle); rPCL (reconstructed PCL)

Similarly, a guidewire is inserted through the AL portal into the femoral tunnel until it exits from the skin on the medial thigh. With the help of a grasper, the loop of the femoral suture is inserted into the tibial loop and retrieved from the tibial tunnel. At this moment, the passing suture connects the exit of the tibial tunnel with the exit of the femoral tunnel, and it could be used to pull the previously harvested graft into the joint (Fig. 8). The author’s preferred fixation methods are suspensory fixation on the femur and screw on the tibia using autologous hamstrings. In comparison, double fixation with screws could be performed if an Achilles tendon allograft or a quadriceps tendon (autograft or allograft) are chosen for the reconstruction. The final fixation is performed by applying an anterior drawer to the knee flexed at 90° with the foot in neutral rotation.

## Conclusion

The PCL reconstruction techniques are continuously evolving over the past years. Using a double PM portal may allow for easier identification of the tibial footprint and more precise and safer placement of the tibial tunnel. Given the only additional theoretical complication risk, it could be considered a valid surgical approach that should be included in the modern PCL surgeon’s armamentarium (Table 1).

**Table 1:** Advantages and limitations

**Advantages:**
Fluoroscopic images and radiation exposure is not necessary
Easier identification of the PCL anatomic tibial footprint
Accurate tibial stump preparation may allow for an easier graft passage avoiding the “killer turn”
Direct guide-pin protection with the aid of a curette introduced from the “working” portal
PM portals could be used to directly visualize the graft passage
No need for additional 70° arthroscope
**Disadvantages**:
Increased theoretical risk of saphenous vein and sartorial branch of the saphenous nerve damage when creating the second portal
Need for a second posteromedial incision

## Abbreviations

PCL: Posterior cruciate ligament
PM: Posteromedial
MFC: Medial femoral condyle
MTP: Medial tibial plateau
AM: Anteromedial
AL: Anterolateral
ACL: Anterior cruciate ligament
